# Relationship closeness buffers the effects of perceived stress on transcriptomic indicators of cellular stress and biological aging marker p16^INK4a^

**DOI:** 10.18632/aging.103739

**Published:** 2020-07-26

**Authors:** Kelly E. Rentscher, Judith E. Carroll, Steve W. Cole, Rena L. Repetti, Theodore F. Robles

**Affiliations:** 1 Cousins Center for Psychoneuroimmunology, Semel Institute for Neuroscience and Human Behavior, University of California Los Angeles, Los Angeles, CA 90095, USA; 2 Department of Psychology, University of California Los Angeles, Los Angeles, CA 90095, USA

**Keywords:** psychological stress, relationship closeness, biological aging, cellular senescence, gene expression

## Abstract

Chronic stress can accelerate biological aging, offering one mechanism through which stress may increase age-related disease risk. Chronic activation of the sympathoadrenal system increases cellular energy production, resulting in cell stress that can initiate cellular senescence, a permanent state of cell growth arrest. Our previous research linked psychosocial stress with increased expression of senescence marker p16^INK4a^; however, less is known about the role of protective psychosocial factors in biological aging. We examined relationship closeness (perceived interconnectedness with one’s spouse) as a protective buffer of the effects of stress on expression of the p16^INK4a^-encoding gene (*CDKN2A*) and transcription control pathways activated under cell stress. Seventy parents (*M*_age_=43.2) completed interview-based and questionnaire measures of psychosocial stress and relationship closeness. Blood samples assessed *CDKN2A* expression and inferred activity of *a priori*-selected transcription factors Nrf2 and heat shock factors (HSFs) via genome-wide transcriptome profiling. Random intercept models adjusting for age, sex, and ethnicity/race revealed that perceived stress was associated with elevated *CDKN2A* expression for parents with low but not high closeness. Secondary bioinformatics analyses linked the interaction of perceived stress and relationship closeness to Nrf2 and HSF-1 activity. Findings identify relationship closeness as a protective factor that may buffer the impact of stress on cellular stress and senescence pathways.

## INTRODUCTION

A growing literature suggests that chronic psychosocial stress exposures can influence biological aging pathways, offering one mechanism through which stress may increase risk for age-related disease. Despite this literature, relatively little is known about protective psychosocial factors that may ameliorate the negative effects of stress on biological aging. High quality and supportive social relationships are associated with reduced physiological stress responses, better overall health, and reduced all-cause mortality. The present study therefore investigated the protection that marital relationships may offer in the face of stress in a sample of midlife parents. We were interested in examining a particular characteristic of marital relationships—relationship closeness, defined as a subjective perception of closeness or interconnectedness with one’s spouse—as a protective factor that may buffer the effects of psychosocial stress on biological aging, because of its potential to influence stress appraisal and coping processes in ways that other aspects of marital quality, such as satisfaction, may not.

Biological aging refers to age-related changes at the molecular, cellular, and intercellular levels, including DNA damage, telomere shortening, cellular senescence, epigenetic modifications, and altered intercellular communication [1]. Age-associated biological changes may serve as mechanisms that contribute to the development of aging phenotypes and diseases such as type 2 diabetes, cardiovascular disease, Alzheimer’s disease, and cancer [2]. A growing literature suggests that chronic psychosocial stress can impact these biological aging pathways [3–5], offering one mechanism through which stress may increase age-related disease risk.

Prolonged or repeated activation of the sympathoadrenal system during stress exposure releases catecholamines that increase cellular energy production and result in cell stress, a state of cellular imbalance in which the production of oxidants exceeds antioxidant capacity [6]. Cells under stress activate compensatory responses to promote detoxification, build antioxidant reserve and respond to cellular injury by activating repair processes. Nuclear transcription factor (TF) Nrf2 is a key regulator of this response, as it plays an important role in cellular responses to oxidative stress [7]. In addition, heat shock factors (HSFs)—particularly HSF-1—regulate the transcription of genes that encode heat shock proteins, which aid in the synthesis, transport, and folding of proteins to protect cells from damage under stressful conditions [8]. Psychological stress has been shown to induce expression of heat shock proteins, demonstrating a role of the sympathoadrenal system in driving cell stress [9]. Unresolved or prolonged cell stress can lead to excess DNA damage and initiate a permanent state of cell growth arrest termed cellular senescence [10, 11]. Whether psychosocial stress exposures lead to cellular senescence is unclear, although our initial cross-sectional findings have linked chronic stress exposure, perceived stress and accumulated daily stress appraisals to increased expression of cell cycle inhibitor p16^INK4a^, a marker of cellular senescence in peripheral blood cells [12].

p16^INK4a^ has been proposed as a biomarker of aging because it correlates highly with chronological age across several tissues in mice and humans [13] and has been implicated in age-related pathologies such as diabetes, cardiovascular disease, and neurodegeneration. Although p16^INK4a^ expression in response to cell stress is thought to prevent the replication of damaged cells that could develop into cancer or other malignancies, pervasive senescence can itself accelerate aging through the release of proinflammatory factors [14]. Critical evidence for the role of senescent cells in age-related disease comes from research demonstrating that removal of p16^INK4a^-positive cells can prevent or slow the deterioration of several tissues and organs, delay tumor growth, and reduce metastasis in mice exposed to cytotoxic cancer treatments [15, 16].

Although research has linked psychosocial stress to several markers of biological aging in humans, causal models that examine the specific mechanisms are lacking. For instance, a sizeable literature suggests that chronic stress exposure over the lifespan is associated with shortened telomere length; yet, only a few studies have prospectively tracked telomere length changes [17]. Several studies have also linked stressors such as caregiving, work-related stress, perceived stress, bereavement, and decreased feelings of closeness with parents during childhood to elevated oxidative stress and DNA damage and repair processes [6, 18, 19]. These studies, combined with our p16^INK4a^ findings, provide support for the hypothesis that chronic psychosocial stress in humans can affect a number of biological aging pathways.

Despite this literature, relatively little is known about protective psychosocial factors that may lessen or even prevent the negative effects of stress on biological aging pathways [18]. Having a higher quality marriage and greater availability of social support is associated with reduced physiological stress responses, including lower cardiovascular reactivity, cortisol reactivity and diurnal profiles, and susceptibility to viral infection and illness [20, 21]. To date, however, studies of aging biology have focused mainly on protective factors in childhood. Specifically, greater parental warmth (assessed retrospectively in adulthood) and parental responsiveness buffered the effects of stress exposure on system-level indicators of biological aging [22], proinflammatory gene expression [23] and telomere shortening [24]. Given the importance of marital relationships for many adults, the limited investigation of protective relationship processes in the context of stress during adulthood represents a gap in knowledge on the role of psychosocial factors in accelerated biological aging as a pathway to disease.

The present study extends this literature by investigating a particular characteristic of marital relationships—relationship closeness—as a protective buffer of the effects of psychosocial stress on two biological aging pathways: gene expression of cellular senescence signal p16^INK4a^ (*CDKN2A*) and transcription control pathways activated under cell stress (Nrf2, HSFs). We were interested in examining relationship closeness as a protective factor because of its potential to influence stress appraisal and coping processes in ways that other aspects of relationship quality, such as satisfaction, may not. For scientists who study intimate relationships, relationship *closeness* and *satisfaction* are conceptualized and studied as separate but related aspects of relationship quality [25]. Closeness refers to the subjective perception of being attached to or interconnected with one’s spouse, whereas relationship satisfaction refers to an attitude or evaluative judgment about the positive and negative features of one’s spouse or relationship. Individuals who perceive a low degree of closeness with their spouse may still have favorable attitudes towards their spouse or relationship. On the other hand, individuals who perceive a high degree of closeness with their spouse may be more likely to view stressful circumstances as shared rather than individual burdens. This may result in access to a greater diversity of resources, particularly their partners’ coping resources, and a more effective set of coping strategies to reduce the impact of stress exposures. To our knowledge, however, no studies have directly tested relationship closeness as a buffer of the negative effects of stress on health.

In addition, whereas previous studies of the stress-buffering effects of parent-child relationships and biological aging have relied on retrospective reports of stress and parental warmth in childhood, this study involves concurrent assessment of psychosocial stress using multiple methods (interview-based, self-report, and intensive repeated measures over 56 days), relationship closeness, and biological aging in a sample of midlife parents. Based on evidence of the stress-buffering effects of high-quality and supportive relationships, we extend our previous findings [12] by hypothesizing that parents who experience less closeness with their spouse will show stronger associations between psychosocial stress and expression of the p16^INK4a^-encoding gene (*CDKN2A*) than those with greater closeness. A secondary aim of the study is to explore upstream transcription control pathways activated under cell stress (Nrf2, HSF-1 and HSF-2); however, the extant literature is not sufficiently developed to inform directional hypotheses.

## RESULTS

### Preliminary analyses

Table 1 presents descriptive statistics and correlations among the five main study variables: chronic stress exposure, perceived stress, accumulated daily stress, relationship closeness, and p16^INK4a^-encoding gene *CDKN2A*. The distribution of chronic stress exposure scores for the sample suggested mild to moderate levels of exposure observed in previous samples [26, 27]. The three psychosocial stress measures were moderately correlated. Chronic stress exposure and accumulated daily stress were negatively correlated with relationship closeness, whereas perceived stress was not. Each of the three stress measures was positively correlated with *CDKN2A* expression, but relationship closeness was not.

**Table 1:** Descriptive statistics and inter-correlations between main study variables (*N* = 70).

**Variables**	***M***	***SD***	**Range**	**1.**	**2.**	**3.**	**4.**	**5.**
1. Chronic stress exposure (1–5)	2.08	0.36	1.27–3.15	-	.44**	.43**	-.24*	.31**
2. Perceived stress (0–40)	12.90	6.35	2.00–32.00		-	.51**	-.12	.38**
3. Accumulated daily stress (%)	21.91	21.16	0–79.63			-	-.29*	.30*
4. Relationship closeness (0–5)	3.17	1.18	0–5.00				-	.01
5. *CDKN2A* expression	6.81	0.04	6.72–6.93					-

*Note*. *p < .05, **p < .01.

Regarding covariates, age was not significantly correlated with *CDKN2A* expression, *r*(70) = .15, *p* = .22. There were no sex differences in *CDKN2A* expression, *t*(68) = -0.77, 95% *CI* for mean difference [-0.03, 0.01], *p* = .45, and *CDKN2A* did not vary as a function of ethnicity/race, *F*(3,65) = 0.70, *p* = .56. Educational status, body mass index (BMI), alcohol use, smoking, upper respiratory infection diagnosis, and medication use were not significantly associated with *CDKN2A* expression (*p*s > .20), nor were the percentage of neutrophil, lymphocyte, monocyte, eosinophil and basophil subsets of total white blood cells (*p*s > .15).

Although they were not related to *CDKN2A* expression, some of the covariates were associated with psychosocial stress and relationship closeness. There were marginal sex differences in perceived stress, *t*(68) = -1.84, *CI* for mean difference [-5.74, 0.24], *p* = .07, with women (*M* = 24.16, *SD* = 6.62) reporting slightly greater stress than men (*M* = 21.41, *SD* = 5.75), and significant sex differences in relationship closeness, *t*(68) = 2.20, 95% *CI* for mean difference [0.06, 1.16], *p* = .03, with men (*M* = 3.50, *SD* = 1.05) reporting greater closeness than women (*M* = 2.89, *SD* = 1.23). BMI was marginally correlated with chronic stress exposure, *r*(69) = .21, *p* = .09. There were marginal differences in perceived stress based on smoking status, *F*(2,66) = 2.59, *p* = .08; post-hoc *t*-tests with Bonferroni correction indicated that parents who smoked 10 or fewer cigarettes per day (*n* = 20; *M* = 25.30, *SD* = 6.86) trended toward higher stress than those who did not smoke (*n* = 46; *M* = 21.67, *SD* = 5.87), *t* = -3.63, *p* = .10. The percentage of eosinophils in the leukocyte pool was marginally correlated with chronic stress exposure, *r*(68) = .24, *p* = .05, and perceived stress, *r*(68) = .22, *p* = .07. Based on these analyses, theoretical considerations, and constraints on power given the sample size, we included age, sex, and ethnicity/race as primary covariates and evaluated smoking status, BMI, and eosinophil percentage as secondary covariates in the models.

### Chronic stress exposure, relationship closeness, and *CDKN2A* expression

An unadjusted random intercept model examined chronic stress exposure, relationship closeness, and their interaction as predictors of *CDKN2A* expression, followed by an adjusted model that accounted for age, sex, and ethnicity/race (Supplementary Table 1). Contrary to expectations, the interaction between chronic stress exposure and relationship closeness on *CDKN2A* expression was not significant in either model.

### Perceived stress, relationship closeness, and *CDKN2A* expression

An unadjusted random intercept model examined perceived stress, relationship closeness, and their interaction as predictors of *CDKN2A* expression, followed by an adjusted model that accounted for age, sex, and ethnicity/race (Table 2). Consistent with hypotheses, there was a significant interaction between perceived stress and relationship closeness on *CDKN2A*. The pseudo R^2^ for the unadjusted model suggested that perceived stress, relationship closeness, and their interaction accounted for approximately 17.8% of the variance in *CDKN2A* expression. Adding covariates to the model slightly reduced the magnitude of the interaction but the interaction coefficient remained statistically significant. Adding BMI and smoking status to the model did not affect the magnitude of the interaction coefficient. Adding eosinophil percentage to the model reduced the magnitude of the interaction coefficient, and the interaction term became marginally significant, *b* = -0.010, *SE* = 0.005, 95% *CI* [-0.020, 0.001], *p* = .07; however, the coefficient for eosinophil percentage itself was nearly zero, *b* = -0.0001, *p =* .98.

**Table 2:** Random intercept models with perceived stress and relationship closeness predicting *CDKN2A* expression (*N* = 70).

**Variables**	**Unadjusted model**					**Adjusted model**			
	***b***	***SE***	***p***	**95% CI**		***b***	***SE***	***p***	**95% CI**
Intercept	6.808	0.005	<.001	[6.798, 6.817]		6.796	0.009	<.001	[6.777, 6.814]
Perceived stress	0.016	0.005	.001	[0.007, 0.026]		0.018	0.005	<.001	[0.009, 0.028]
Relationship closeness	0.003	0.005	.47	[-0.006, 0.013]		0.002	0.005	.68	[-0.008, 0.012]
Perceived stress **×** Relationship closeness	-0.012	0.005	.02	[-0.023, -0.002]		-0.011	0.005	.04	[-0.021, -0.001]
Age						0.006	0.005	.22	[-0.004, 0.016]
Sex						-0.002	0.010	.84	[-0.023, 0.019]
Ethnicity/race						0.005	0.003	.09	[-0.001, 0.011]

*Note*. CI = confidence interval. All continuous variables were *z*-transformed.

**Figure 1:**
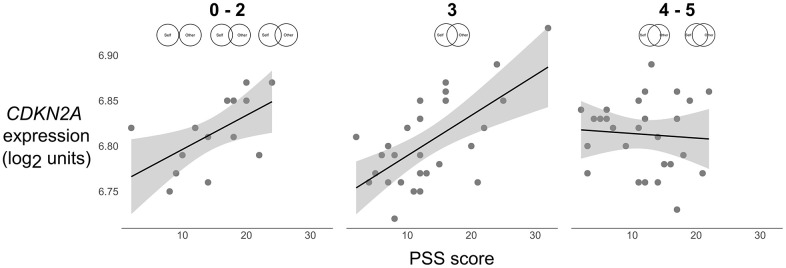
**Scatterplots of the association between perceived stress (PSS) and expression of the p16^INK4a^-encoding gene *CDKN2A* (log_2_ units) at low (0–2), average (3), and high (4–5) levels of relationship closeness (scale item responses depicted above each plot).** Solid lines were plotted using parameter estimates from the unadjusted models in Table 2. Grey shaded bands reflect the 95% CI for the best fit regression line computed from the raw data.

Follow-up analyses that probed the interaction for the adjusted model revealed that for individuals with low (*simple slope* = 0.030, *SE* = 0.007, *p* < .001) and moderate (*simple slope* = 0.019, *SE* = 0.005, *p* < .001) relationship closeness, greater perceived stress was associated with elevations in *CDKN2A* expression (Figure 1). In contrast, for individuals with high (*simple slope* = 0.007, *SE* = 0.007, *p* = .30) relationship closeness, the association between perceived stress and *CDKN2A* was not significant. The region of significance for the interaction was below 0.63 *SD* on relationship closeness, suggesting that individuals who endorsed a 3.91 or higher on the relationship closeness scale (i.e., 4 or 5 on a 0–5 scale) showed a stress-buffering effect of relationship closeness.

To test an alternative hypothesis that relationship satisfaction might account for the stress-buffering effect of relationship closeness, an additional random intercept model examined perceived stress, relationship satisfaction, and their interaction as predictors of *CDKN2A* expression, followed by an adjusted model that accounted for age, sex, and ethnicity/race. As expected, the interaction term was not significant in the unadjusted (*b* = -0.006, *SE* = 0.005, 95% *CI* [-0.016, 0.004], *p* = .24) or adjusted (*b* = -0.004, *SE* = 0.005, 95% *CI* [-0.014, 0.006], *p* = .44) models. In a final analysis that included relationship satisfaction as an additional covariate in the model with perceived stress, relationship closeness, and their interaction as predictors of *CDKN2A* expression, the perceived stress x relationship closeness interaction term remained significant, *b* = -0.012, *SE* = 0.005, 95% *CI* [-0.022, -0.001], *p* = .03. A finding suggesting that relationship closeness has a unique impact on *CDKN2A* expression is noteworthy given that relationship closeness and satisfaction were highly correlated, *r*(70) = .50, *p* < .001.

### Accumulated daily stress, relationship closeness, and *CDKN2A* expression

An unadjusted random intercept model examined accumulated daily stress, relationship closeness, and their interaction as predictors of *CDKN2A* expression, followed by an adjusted model that accounted for age, sex, and ethnicity/race (Supplementary Table 2). Contrary to expectations, the interaction between accumulated daily stress and relationship closeness on *CDKN2A* expression was not significant in either model.

### Transcription control pathways activated under cell stress

**Figure 2:**
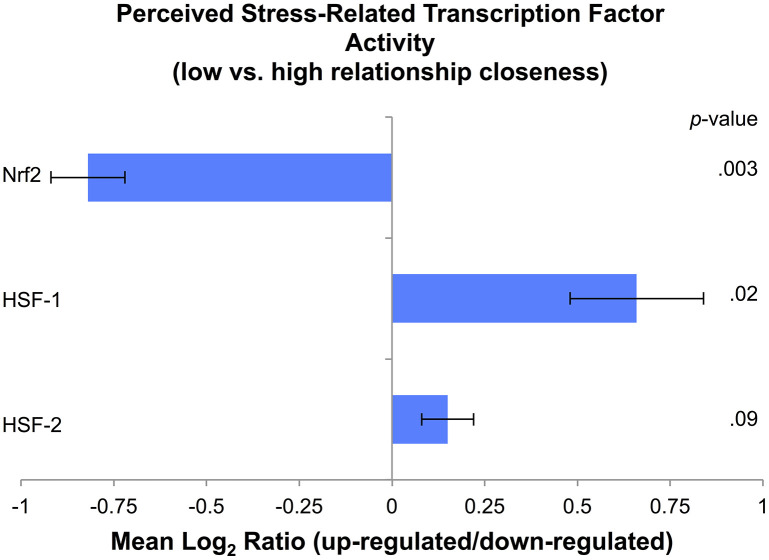
**Cell stress transcription factor activity based on levels of perceived stress in parents with low (scores of 0–3 on the IOS scale) relative to high (scores of 4–5 on the IOS scale) relationship closeness, expressed as a Mean Log_2_ Ratio of transcription factor binding motif prevalence in the promoter regions of up-regulated versus down-regulated genes, averaged across nine parametric variations.**

Bioinformatics analyses assessed the inferred activity of three *a priori*-selected TFs activated under cell stress: Nrf2, HSF-1, and HSF-2. Based on findings from the main analyses, these secondary analyses focused on the interaction between perceived stress and relationship closeness (low: scores of 0–3 on IOS; high: scores of 4–5 on IOS, based on the region of significance in the main analysis), with age, sex, and ethnicity/race entered as covariates. Analyses identified gene transcripts with a point estimate of greater than 1.2-fold for the interaction of perceived stress and relationship closeness. A total of 50 differentially expressed genes (Supplementary Table 3) that showed significant up-regulation (25 genes) and down-regulation (25 genes) were then submitted to TELiS to assess the likelihood of whether the TF binding motifs were over- or under-represented in the promoter regions of genes up-regulated in association with the interaction profile. Among parents who reported less relationship closeness, relative to those with greater closeness, perceived stress was associated with reduced Nrf2 representation (*Mean Log_2_ Ratio [MLR]* = -0.82, *SE* = 0.10, *p* = .001; across all 9 parametric variations; Figure 2). Among parents who reported less relationship closeness, relative to those with greater closeness, perceived stress was associated with greater HSF-1 (*MLR* = 0.66, *SE* = 0.18, *p* = .02; 5 out of 9 parametric variations) and marginally greater HSF-2 (*MLR* = 0.15, *SE* = 0.07, *p* = .09; 6 out of 9 parametric variations) representation.

### Exploratory follow-up analysis

An exploratory follow-up analysis examined potential sex differences in the interaction of perceived stress and relationship closeness on expression of the p16^INK4a^-encoding gene *CDKN2A*. In an adjusted random intercept model that accounted for age and ethnicity/race, the 3-way interaction between perceived stress, relationship closeness, and sex on *CDKN2A* expression was not statistically significant, *b* = 0.010, *SE* = 0.012, 95% *CI* [-0.014, 0.034], *p* = .41. When the analyses were stratified by sex, random intercept models that accounted for age and ethnicity/race revealed that the interaction between perceived stress and relationship closeness on *CDKN2A* expression was marginally significant for male participants, *b* = -0.018, *SE* = 0.009, 95% *CI* [-0.036, 0.0004], *p* = .06, but not significant for female participants, *b* = -0.008, *SE* = 0.007, 95% *CI* [-0.022, 0.006], *p* = .27. It is important to note that because the exploratory stratified analyses are underpowered and do not account for potential interdependence of the data between relationship partners, they are interpreted with caution.

## DISCUSSION

The present study investigated midlife parents’ relationship closeness as a protective factor that can buffer the effects of psychosocial stress—assessed via interview-based, questionnaire, and daily diary measures—on expression of the p16^INK4a^-encoding gene *CDKN2A* and upstream transcription control pathways activated under cell stress (Nrf2, HSF-1, HSF-2). As hypothesized, parents who perceived their lives as more stressful in the week prior to study entry showed elevations in *CDKN2A* expression—but only when they reported less closeness with their spouse. In contrast, parents who reported a high degree of closeness, or sense of interconnectedness with their spouse, did not show stress-related elevations in *CDKN2A*. As one of the most robust indicators of cellular senescence, p16^INK4a^ signals a permanent state of cell growth arrest. This finding has potentially important implications for health, as senescent cells have been associated with reduced stem cell and tissue function and increased proinflammatory factors that are thought to contribute to age-related disease and functional declines.

Importantly, a follow-up analysis testing an alternative hypothesis that relationship satisfaction might account for the observed effect revealed that the stress-buffering effects on expression of the p16^INK4a^-encoding gene *CDKN2A* were unique to relationship closeness, independent of perceived relationship satisfaction. Although the present study did not directly test the psychological mechanisms involved, this finding is consistent with the hypothesis that relationship closeness may uniquely buffer the effects of stress on health by influencing stress appraisal and coping processes in ways that other aspects of relationship quality—such as relationship satisfaction—may not. Future research may benefit from testing specific mechanisms, such as whether individuals who perceive a high degree of closeness with their spouse are more likely to view stressors as shared rather than individual burdens and more readily draw on their partners’ available coping resources in addition to their own to reduce the impact of a stressor and buffer the physiological stress response.

Although previous research has shown that cell distribution can influence estimates of gene expression [13], cell subset percentages did not relate to *CDKN2A* expression in this study. However, parents with higher perceived stress did show a marginally higher proportion of eosinophils in the leukocyte pool, and accounting for eosinophil percentage in the main analyses reduced the interaction between perceived stress and relationship closeness on *CDKN2A* to marginal significance. As eosinophils are a source of oxidative stress [28], their mobilization and activation may play a role in the mechanistic pathway through which psychosocial stress leads to increased cell stress, and ultimately, cellular senescence. Stress-related changes in cell distribution and how they relate to markers of biological aging is an important area for future investigation.

Secondary bioinformatics analyses revealed that, relative to parents who experienced greater closeness with their spouses, those with less closeness showed reduced stress-related Nrf2 representation. Nrf2 is considered a key TF for protecting the cell against oxidative stress, as it regulates genes that encode detoxifying enzymes and antioxidant proteins [7]. In addition, relative to parents who experienced greater closeness, those with less closeness showed greater stress-related HSF-1 representation and marginally greater HSF-2 representation. Previous research has found that HSFs regulate genes that encode heat shock proteins, which aid in the synthesis, transport, and folding of proteins under conditions of stress [8]. This pattern of findings suggests that parents with low closeness may show a reduced antioxidant response under conditions of oxidative stress that could contribute to accumulated cellular damage and lead to cellular senescence, whereas parents with high closeness show a robust anti-oxidant response that could protect against cellular damage. Although these cross-sectional data cannot address whether Nrf2 and HSF activation are a direct compensatory response to cell stress, or whether they are activated simultaneously or sequentially, findings suggest plausible associations with these mechanisms that warrant investigation in future research.

It was somewhat unexpected that relationship closeness did not buffer the effects of chronic stress exposure and accumulated daily stress on *CDKN2A* expression. It is interesting to note, however, that whereas perceived stress was not correlated with relationship closeness, chronic and daily stress showed small to medium associations with relationship closeness. The lack of correlation with perceived stress is somewhat surprising given the potential shared method variance between the two measures (i.e., both involve single-occasion reporting that is more reflective or evaluative). Nonetheless, although we were unable to test directionality, these associations raise questions about the influence of relationship closeness on the intensity and frequency of stressors. For instance, whether being in a relationship characterized by high closeness might reduce daily parenting stress burden or prevent exposure to certain forms of chronic stress—and how this relates to biological aging—remains to be tested in future research. Although it is possible that greater statistical power may have been required to detect interactions involving chronic and accumulated daily stress due to their correlations with relationship closeness, our findings suggest that the stress-buffering effects of relationship closeness on biological aging may be specific to appraisals that are more reflective or evaluative in nature (e.g., global perceptions of stress over the previous week).

Findings from this study should be considered in light of several limitations. Most notably, the relatively small sample size reduced the statistical power to detect potential moderating effects of sex and ethnicity/race, although exploratory follow-up analyses suggested a potential sex difference in which male participants may experience slightly greater stress-buffering effects than female participants. Future research should investigate these moderators in larger cohorts. Second, although the study included measures of stress that spanned several months, the single-occasion measurement of relationship closeness and gene expression precluded the investigation of directionality. Prospective longitudinal designs that include repeated assessments will be important in future work to address questions about the relative contribution of psychosocial risk and protective factors to rates of biological aging over time. Third, as estimates of *CDKN2A* expression for the study were derived from a single blood sample for each participant, it will be important for future research to address the reliability of *CDKN2A* expression measurement in humans. Fourth, it was somewhat surprising that participants’ chronological age and *CDKN2A* expression were not correlated in this study, given that a previous population-based study reported a significant correlation [13]. This may be due in part to the small sample size and restricted age range of the present sample (*M* = 43.2 ± 7.0, range: 27.6–61.9 years), which was nearly half the range in the previous study (Liu et al. [2009] included adults aged 18-80 years), and it is possible that an association may have been observed had the present sample included older adults. Regardless, the lack of association with age is consistent with the hypothesis that psychosocial stress may contribute to accelerated or premature cellular senescence (i.e., that is not age-associated) during middle adulthood; however, future research to address this question is warranted. On a related note, the lack of association between *CDKN2A* expression and both BMI and smoking status was somewhat surprising, as they are indicators of poor health outcomes; however, previous research has also failed to detect associations [13].

Despite these limitations, the present study extends the literature on psychosocial factors on biological aging in several important ways. First, it is the first to demonstrate that close relationships in adulthood can provide protection against the negative effects of psychosocial stress and biological aging. Findings are consistent with a cellular stress-senescence pathway in which psychosocial stress increases cell stress and damage that, if unresolved, leads to cellular senescence [6, 11]. Although previous research has found that psychosocial stress can impact this pathway, few studies have examined whether protective psychosocial factors can modify these processes, either by reducing the impact of stress on damaging allostatic mechanisms or strengthening restorative repair mechanisms in response to damage. This study provides preliminary evidence that this pathway can be modulated by the quality of marital relationships, which is an exciting avenue for future research. Second, findings suggest that relationship closeness can have unique stress-buffering effects on cellular senescence that are independent of other aspects of relationship quality such as satisfaction. Third, to our knowledge, this is the first study to link psychosocial factors to inferred activity of upstream transcription control pathways activated under cell stress. Pending replication in future studies, relationship closeness is a concrete behavioral target that has the potential to inform the development of psychosocial interventions for parents who may be at risk for stress-related accelerated aging during this life stage.

## MATERIALS AND METHODS

### Participants

Participants were 70 adults from 40 heterosexual couples (54.3% self-reported female) with a mean age of 43.2 years (*SD* = 7.0) and at least one child aged 8–13 years. Participants self-identified as White/Non-Hispanic (44.3%), Hispanic/Latinx (22.9%), Black/African American (18.6%), Asian (12.9%), and Native American/Alaskan Native (1.4%). All participants had a high school diploma or equivalent (42.8%), bachelor’s degree (34.3%), or master’s, professional, or doctoral degree (22.9%). The majority was employed full-time (61.4%), followed by part-time (14.3%) or as homemakers (12.9%). Most participants were married or in a marriage-like relationship (97.1%), with an average relationship length of 15.7 years (*SD* = 5.8). Participants were recruited as part of a larger study on the effects of the family environment on immune function and risk for upper respiratory infection [29, 30]. Families were recruited in the Los Angeles area from 2009 to 2012 through advertising in local elementary and middle schools, libraries and recreation centers, medical clinics, newspapers, and direct mailings using a marketing list of families within five miles of the University of California, Los Angeles, that were selected based on zip-code level income.

### Procedures

At study entry, participants completed an interview-based assessment of chronic stress exposure, followed by questionnaires to assess their perceived stress and their marital relationship quality. During a subsequent 56-day diary period, participants completed online surveys each evening before bedtime that included items related to their daily stress appraisals. At the end of the diary period, participants provided a blood sample that was used to assess gene expression of cellular senescence marker p16^INK4a^ and inferred activity of cell stress TFs. If participants reported any of the following symptoms on the day of the blood draw, the blood draw was rescheduled for a later date: cold or flu-like symptoms such as sore throat, runny nose, or cough, a fever, night sweats, nausea, vomiting, or diarrhea, blood in stool or urine, frequent urination, and/or a skin rash or abscess. There were no restrictions on what participants could consume prior to the draw. Blood samples were collected between 12 p.m. and 7 p.m. at the UCLA Clinical Laboratory through antecubital venipuncture in PAXgene Blood RNA tubes (Qiagen) and transported to the UCLA Health Psychology Laboratory for storage at –80**°** C. Given that the daily diary protocol involved a significant time commitment for participants, the blood sample was not required for participation in the study; 73 of the 86 enrolled adult participants chose to complete the blood draw. Participants who provided blood samples did not differ significantly in their age, sex, education, employment status, income, or BMI from those who did not provide samples. The final sample was further reduced to 70 participants who also completed reports of perceived stress and relationship closeness. Informed consent has been obtained from all participants. Data that support the findings of this study are available upon request from the corresponding author, but are not publicly available due to privacy or ethical restrictions.

### Measures

### Psychosocial stress measures

**Chronic stress exposure.** Participants were administered the 60- to 90-minute semi-structured UCLA Life Stress Interview [31], which was designed to assess exposure to chronic stressors in thirteen domains (e.g., family relationships, friendships, work, finances, health) over the past six months, independent of participants’ subjective appraisals or emotional reactions to the stressors. Trained interviewers asked a series of open-ended questions, with additional probes as necessary to obtain sufficient information to score each domain from 1 (exceptionally good conditions) to 5 (extreme adversity). The present study used an adapted version of the interview that included questions that specifically asked about conflict and warmth in the marital (e.g., “Do you ever argue or fight with your spouse?”) and parent-child (e.g., “How do you feel about your time together with your child?”) relationships. Ratings for the thirteen domains were averaged to create a total score, with higher scores indicating greater exposure.

**Perceived stress.** Participants completed the 10-item Perceived Stress Scale [26], a well-established and validated measure of the degree to which an individual appraises their life as stressful. Items on the scale assess different aspects of perceived stress, including feeling stressed, upset, or angry, and unable to cope with or control important things in life, which participants rated on a scale from 0 (never) to 4 (very often) over the previous week. Items were summed to create a total score, with higher scores indicating greater stress.

**Accumulated daily stress appraisals.** During the 56-day diary period, participants provided reports of daily stress appraisals by rating how accurately the adjectives “stressed” and “overwhelmed” described how they felt that day on a scale from 1 (completely inaccurate) to 4 (completely accurate). In order to assess the accumulation of stress appraisals over the 56-day period, we created a categorical variable in which responses of a “3” (mostly accurate) or “4” (completely accurate) on either of the two items for a given day represented a stress appraisal for that day. We then calculated the total number (sum) of stressful days for each participant over the 56-day period, divided by the number of diary days each participant completed, and multiplied by 100 to create a percentage score. The average participant completed 52.69 (*SD* = 7.24) out of 56 daily diaries.

### Relationship measures

**Relationship closeness.** Participants completed a modified version of the Inclusion of Other in Self (IOS) scale [32], a single-item measure of an individual’s perceived closeness with their spouse that assesses the degree to which one’s partner is perceived as part of one’s self. The IOS scale depicts a set of Venn-diagrams with circles that overlap to varying degrees and create an interval-like scale ranging from 0 (no overlap) to 5 (almost complete overlap). Participants selected the pair of circles that best described their relationship with their spouse.

**Relationship satisfaction.** Participants completed the Couples Satisfaction Index [33], a 32-item measure of an individual’s satisfaction in their romantic relationship that was developed using item response theory with a pool of items from several relationship satisfaction measures. The response scale for the first item (“Please indicate the degree of happiness, all things considered, of your relationship.”) ranges from 0 (extremely unhappy) to 6 (perfect), and the response scale for the remainder of the items ranges from 0 to 5, with anchors specific to sets of items. All items were summed to create a total score, with higher scores indicating greater relationship satisfaction. The average score was 120.76 (*SD* = 27.10) out of 200.

### Gene expression measures

RNA was extracted from the peripheral blood samples (Qiagen RNeasy), tested for suitable mass (Nanodrop ND1000) and integrity (Agilent Bioanalyzer) and converted to fluorescence-tagged cRNA (Ambion TotalPrep). RNA samples were assayed in a single batch using microarray-based genome-wide transcriptome profiling (Illumina Human HT-12 v4 BeadArrays) following the manufacturer’s standard protocol in the UCLA Neuroscience Genomics Core Laboratory. All samples yielded valid results according to standard quality assurance methods (e.g., median probe fluorescence intensity > 100 units) [30].

**Cellular senescence marker p16^INK4a^.** For the present analyses, estimated mRNA levels of the p16^INK4a^-encoding gene *CDKN2A* served as the measure of cellular senescence. Previous research has identified p16^INK4a^ -induced cellular senescence as a permanent state of cell cycle arrest that is not reversible within the cell [1, 11], suggesting that p16^INK4a^ levels are fairly stable. Therefore, the assessment of expression of the p16^INK4a^-encoding gene *CDKN2A* in this study is an estimate of the number of senescent cells in circulation at the time of the blood draw.

**Cell stress transcription control pathways.** Genome-wide transcriptome profiling was used to assess the inferred activity of three *a priori*-selected TFs that previous research has found to be activated under cell stress—nuclear factor erythroid 2–related factor 2 (Nrf2) and HSFs (HSF-1 and HSF-2)—using bioinformatics analyses described in Section 4.6.

### Covariates

Several variables that might affect the number of leukoctyes in circulation and estimates of *CDKN2A* expression were evaluated as potential covariates in the main analyses based on previous research [12, 29, 34]. Variables included age, sex, ethnicity/race, educational status, BMI (kg/m^2^; *M* = 27.90, *SD* = 5.61), average number of alcoholic drinks per week (*M* = 2.56, *SD* = 3.57) and smoking assessed during diary period: none (65.7%), fewer than 10 cigarettes per day (28.6%), or more than 10 cigarettes per day (4.3%), and whether participants met criteria for an upper respiratory infection at any point during the 56-day diary period (30.0%). We also evaluated whether participants were taking medication to treat medical conditions such as hypertension, inflammatory conditions, hypothyroidism, depression, anxiety, and attention-deficit/hyperactivity disorder. Potential covariates also included the percentage of neutrophil (*M* = 56.04, *SD* = 8.46), lymphocyte (*M* = 33.78, *SD* = 7.23), monocyte (*M* = 7.37, *SD* = 2.07), eosinophil (*M* = 2.31, *SD* = 1.70), and basophil (*M* = 0.39, *SD* = 0.47) subsets of total white blood cells, as variations in leukocyte composition may influence the estimation of mRNA [13]. Cell subsets were obtained by complete blood count with differential assessed by the UCLA Clinical Laboratory and Pathology Services using standard clinical laboratory methods.

### Data analysis

All continuous predictor variables and covariates were standardized (*z*-transformed) and gene expression data were quantile-normalized [35, 36] and log2-transformed prior to analysis. Because the sample was composed of 70 adults nested within 40 dyads, including two members of a couple in an ordinary least squares regression model would violate statistical assumptions of independence. The intraclass correlation coefficient for *CDKN2A* expression was *r*(30) = .11, *p* = .57, suggesting that a very small proportion of the variation in *CDKN2A* was accounted for by the particular dyad in which a person was nested. However, because we expected that partners’ psychosocial stress or relationship closeness scores might be more highly correlated, which could introduce another potential source of non-independence in the data, we conducted a series of random intercept models using the mixed procedure in SPSS (version 25) to account for the nesting of participants in dyads [37]. As recommended by Kenny, Kashy, and Cook [38], models used restricted maximum likelihood with a compound symmetry covariance structure to estimate fixed effects and random intercepts. Slopes were constrained to be equal across dyads (i.e., the random component for slopes was omitted from the models), as there are not sufficient lower units to allow slopes to vary across dyads.

Preliminary analyses examined correlations among the main study variables, as well as between the main variables and potential covariates for inclusion in the main analyses. To test the main hypotheses, we conducted a set of unadjusted random intercept models to examine stress, relationship closeness, and their interaction as predictors of *CDKN2A* expression, followed by a set of adjusted models that accounted for covariates. To estimate the amount of variance in *CDKN2A* expression that was accounted for by stress, relationship closeness, and their interaction, we calculated a pseudo R^2^ value using the formula R^2^ = 1 – [(ss_d_ + ss_e_^2^)/(ss_d_′ + ss_e_^2^′)] where ss_d_ is the dyad covariance and ss_e_^2^ is the error covariance derived from the conditional model and the prime indicates covariance derived from the unconditional model [38]. Follow-up analyses used an online computational tool for probing interaction effects in mixed models [39] to provide point estimates for simple slopes representing the association between stress and *CDKN2A* expression at low (-1 SD), moderate (mean), and high (+1 SD) levels of relationship closeness, as well as the region of significance for the interaction effect.

To test an alternative hypothesis that relationship satisfaction might account for the stress-buffering effects of relationship closeness on *CDKN2A* expression, we performed a second set of random intercept models. First, an unadjusted model examined stress, relationship satisfaction, and their interaction as predictors of *CDKN2A* expression, followed by an adjusted model that included covariates. Finally, a follow-up analysis included relationship satisfaction as an additional covariate in the random intercept model with stress, relationship closeness, and their interaction as predictors of *CDKN2A* expression, to test the unique contribution of relationship closeness (independent from satisfaction) in predicting *CDKN2A* expression.

To assess the inferred activity of the three *a priori*-selected TFs activated under cell stress (Nrf2, HSF-1, HSF-2), secondary analyses adopted a promoter-based bioinformatics approach. The list of the differentially expressed genes that had a point estimate of greater than 1.2-fold for the interaction of perceived stress and relationship closeness were entered into the Transcript Element Listening System (TELiS) [40]. The 1.2-fold threshold is consistent with prior studies linking psychosocial factors to gene expression [23, 41]. TELiS contains data on the prevalence of 192 TF binding motifs from the TRANSFAC database [42]; however, the present analysis focused on a pre-specified set of three TF binding motifs (Nrf2, HSF-1, HSF-2) based on *a priori* hypotheses related to cellular stress. TELiS analyses involve a test of the log ratio of TF binding motif prevalence in the promoter regions of up-regulated versus down-regulated genes, with results averaged across nine parametric variations of promoter sequence length (–300 base pairs [bp] upstream of the RefSeq gene transcription start site, –600 bp, and –1000 to +200 bp) and TF binding motif match stringency (Transfac mat_sim values ≥ .80, .90, and .95), and standard errors derived by bootstrapping of residuals (200 cycles of resampled residual vectors, which controls for any potential correlation among residuals across genes) [40, 43]. Activation of Nrf2 was indicated by the TRANSFAC V$NRF2_01 DNA motif and activation of the HSFs was indicated by V$HSF1_01 and V$HSF2_01.

### Ethics statement

This investigation has been conducted in accordance with the ethical standards and according to the Declaration of Helsinki and according to national and international guidelines, and has been approved by the Institutional Review Board at the University of California, Los Angeles.

## Supplementary Material

Supplementary Tables
