# Supplementary material for: Relationship closeness buffers the effects of perceived stress on transcriptomic indicators of cellular stress and biological aging marker p16INK4a
Source: Aging (Albany NY). 2020 Jul 26;12(16):16476–90. doi: 10.18632/aging.103739 (PMC7485710; doi:10.18632/aging.103739)
Supplement: Supplementary Tables [file aging-12-103739-s001..pdf]

SUPPLEMENTARY TABLES

Supplementary Table 1. Random intercept models with chronic stress exposure and relationship closeness predicting *CDKN2A* expression (*N* = 70).

Variables	Unadjusted model				Adjusted model			
	b	***SE***	***p***	**95% CI**	***b***	***SE***	***p***	**95% CI**
Intercept	6.808	0.005	<.001	[6.798, 6.819]	6.797	0.010	<.001	[6.777, 6.817]
Chronic stress	0.014	0.005	.01	[0.004, 0.025]	0.014	0.005	.01	[0.004, 0.025]
Relationship closeness	0.005	0.005	.38	[-0.006, 0.016]	0.004	0.006	.52	[-0.008, 0.015]
Chronic stress × Relationship closeness	-0.003	0.004	.46	[-0.012, 0.006]	-0.001	0.005	.84	[-0.010, 0.008]
Age					0.005	0.005	.31	[-0.005, 0.016]
Sex					0.004	0.011	.70	[-0.018, 0.026]
Ethnicity/race					0.004	0.003	.27	[-0.003, 0.010]

Note. CI = confidence interval. All continuous variables were *z*-transformed.

Supplementary Table 2. Random intercept models with accumulated daily stress and relationship closeness predicting *CDKN2A* expression (*N* = 70).

Variables	Unadjusted model				Adjusted model			
	b	***SE***	***p***	**95% CI**	***b***	***SE***	***p***	**95% CI**
Intercept	6.808	0.005	<.001	[6.798, 6.819]	6.800	0.010	<.001	[6.780, 6.821]
Daily stress	0.013	0.005	.02	[0.002, 0.024]	0.012	0.006	.04	[0.001, 0.023]
Relationship closeness	0.005	0.005	.38	[-0.006, 0.016]	0.004	0.006	.47	[-0.007, 0.016]
Daily stress × Relationship closeness	-0.002	0.004	.67	[-0.011, 0.007]	-0.001	0.005	.78	[-0.011, 0.008]
Age					0.004	0.005	.50	[-0.007, 0.015]
Sex					0.004	0.011	.70	[-0.018, 0.026]
Ethnicity/race					0.002	0.003	.49	[-0.004, 0.009]

Note. CI = confidence interval. All continuous variables were *z*-transformed.

Supplementary Table 3. List of differentially expressed genes (>1.2-fold) for the interaction of perceived stress and relationship closeness (low vs. high).

Up-regulated genes	Down-regulated genes
LOC728937	HLA-A29.1
LOC100131971	TUBB2A
CCL3L3	FCGR1B
LOC100129650	LILRA3
TMEM158	FCGR1A
SERPINA13	FCGR1C
CFD	ORM1
HBG2	LOC100133875
IFI27	PI3
ACCS	HS.137971
LOC728823	HLA-DQB1
LOC644191	GNG10
LOC641768	HS.508682
LOC645979	IL18RAP
LOC441377	SIGLEC14
LOC650646	SLPI
RPS26P11	ANXA3
LOC644934	CXCL10
MYOM2	C19ORF59
LOC644928	LOC731682
RPS26L	PROK2
RPS26	SCGB3A1
FOLR3	S100A12
HLA-DRB1	DEFA1
HLA-DRB5	TNFSF13B
